# Novel Technique for Backside Alignment Using Direct Laser Writing

**DOI:** 10.3390/mi16030255

**Published:** 2025-02-25

**Authors:** Melissa Mitchell, Siva Sivaraya, Simon J. Bending, Ali Mohammadi

**Affiliations:** 1 Department of Electronic and Electrical Engineering, University of Bath, Claverton Down, Bath BA2 7AY, UK; 2 Department of Physics, University of Bath, Claverton Down, Bath BA2 7AY, UK

**Keywords:** alignment, MEMS, microfabrication, nanofabrication

## Abstract

Backside alignment is a key microfabrication process step, especially in micro-electromechanical systems (MEMS). The double-side mask aligners used for this purpose are unaffordable for many research centres. We propose a new method that aligns the backside mask to the features on the topside using a direct laser writer, which is available in many cleanrooms. In this method, the corner co-ordinates of the sample are used as alignment features, and a transformation matrix is developed to map the design co-ordinates to the stage co-ordinates. This method has been validated on copper features as small as 100 μm on silicon substrates. Test samples are cut from a 2 inch Si wafer, and copper features are sputtered and developed onto the topside. Backside patterns that are aligned to these copper features are created using photolithography through the application of this alignment method. This method exhibited challenges for samples without sharp right-angled corners, where the estimation of the corner co-ordinates resulted in misalignment. Sixteen areas over nine samples were analysed. An average alignment resolution of 23 ± 1 μm was established in the x and 8 ± 4 μm in the y direction, and a rotation misalignment of less than 1° was achieved. Differences in alignment were due to the individual quality of each sample’s corners and to the clarity of the corner co-ordinates. This new approach provides a route towards low-cost microfabrication process development.

## 1. Introduction

The fabrication of micro-electromechanical systems (MEMS) on silicon wafers often requires lithographic procedures on both the front and backside of the wafer. For example, etching cavities for pressure sensors that release the active part of the transducer [1], microfluidic structures with metasurface cavities for the improved interaction of sample and incident radiation [2], or thinning microheater substrates for reducing heat loss [3]. It is, therefore, vital that feautures on both sides can be aligned. Current backside alignment methods rely heavily on specialist equipment or infrared imaging, which is either unavailable to many research labs or increases the cost of the microfabrication process.

As many semiconductor substrate materials, including silicon, are transparent against infrared (IR) light, commonly, an infra-red camera is used that allows for the visualisation of both sides of the chip simultaneously [4]. However, the IR cameras needed for imaging both sides of a silicon wafer simultaneously as part of a high precision alignment system are expensive and unavailable in most research laboratories. Furthermore, only some substrates, such as silicon, are transparent to IR; hence, this technique does not apply to printed circuit boards (PCBs) or substrates plated with metallic layers.

Nikon offer a contactless pre-alignment system that allows for a higher accuracy of over 500 nm when loading a wafer onto the stage. This method requires notch orientation prior to loading, and a fine alignment after the wafer is loaded onto the wafer chuck [5]. Similarly, the Nikon i-line Stepper NSR-SF150 has a non-contact infrared alignment system that can use embedded alignment marks on the wafer to align the wafer stacks. This technique allows for a misalignment smaller than 200 nm in x and y directions [6]. However, both of these methods require expensive specialist equipment that is not readily available in most nano-fabrication labs.

Many of the systems that allow front-to-backside alignment have limitations for the size of the samples; some require either 2 or 4 inch silicon wafers, which is not suitable for samples with arbitrary sizes that are commonly used for research purposes. For example, Suss MA300 accommodates 200 mm and 300 mm samples, and the EVG620 double side mask aligner is only suitable for samples with diameters ranging from 50 mm to 150 mm.

Alternatively, simpler methods have been suggested that mitigate the need for expensive double-sided mask aligner systems. Alignment can be achieved using a single-side mask aligner, which is generally available even in smaller laboratories with more limited facilities. This technique utilises external alignment marks (EAM) on a transparent substrate, which can be seen from both sides of the sample, while the marks on the silicon wafer can only be seen from the frontside. A thin cover glass is attached to the substrate for this purpose. This technique offers an accuracy of 8–11 μm and precision of 3 μm [7].

Some methods require further equipment, although they are not as expensive as double-side mask aligners or IR systems. A mechanical jig has been proposed that requires two masks for only the alignment marks of both sides of the wafer. This method has a theoretical accuracy, and is expected to have an alignment error totaling less than 1 μm, and requires the alignment marks be aligned with the crystallographic structure of the wafer [8].

A similar method also utilises a mechanical jig for the formation of a set of reference marks on both sides of the wafer, which are mutually aligned. This setup achieves an alignment accuracy of around 4–5 μm and cannot be used for irregular-shaped wafers [9].

A projector is another potential option for generating fiducial marks on one side of the wafer, which are then used for aligning the patterns on both sides of the wafer. This method claims an accuracy of around 1 μm [10].

Mask modification has also been suggested, which uses square-shaped ‘reference frames’ that are created on both of the mask plates. The patterns are found within this reference frame, with the corners of the square being used as the origin co-ordinates. The flat side of the wafer is aligned with the frame. This provides an alignment accuracy of 2–4 μm, but it cannot be used if there is no wafer flat, or if the wafer flat is distorted [11].

Some more recent developments include using a Nikon alongside a Suss mask aligner to deliver sub-micron alignment accuracy, although this, again, requires expensive equipment [12]. Another method has been suggested using multiple lithographic exposures, although this is very time-consuming there are no alignment accuracy figures states [13].

Nearly all of these methods rely on expensive alignment instruments or additional equipment to be made in-house, and most of them limit the material, size, or shape of the sample that can be used. This paper presents an innovative technique to use a direct laser writer (DLW) for backside alignment. DLWs are readily available to many microfabrication facilities at a moderate cost. The new method proposed in this work can be used with any material or sample size, requires no additional equipment, and has an accuracy of the same order of magnitude as some of the more complex methods.

Section 2 outlines the theory of the proposed backside alignment method and details the experimental steps. Section 3 reviews the results, including a comparison of the proposed method against the existing systems in Table 1.

## 2. Materials and Methods

### 2.1. Proposed Backside Alignment Technique

#### 2.1.1. Direct Laser Writer (DLW)

Direct laser writing is a photolithography technique that utilises UV lasers to locally expose photoresist; the focused laser beam is used to map structures and designs in photoresist [14]. These multipurpose systems are commonly used in prototyping microdevices without the use of a mask, or can also be used to create a permanent glass mask for use with other systems, such as mask aligners.

**Figure 1:**
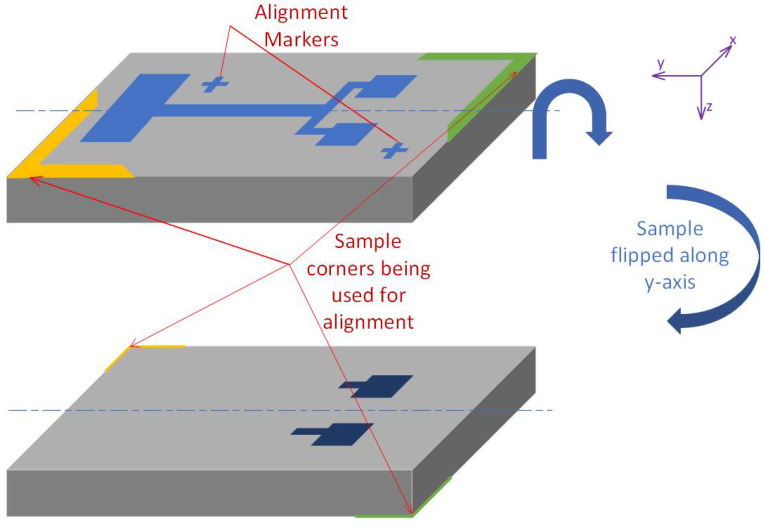
Illustration of how the corners and alignment marks will be affected by the sample being flipped when replaced on the stage following photoresist spinning onto the backside.

DLWs have alignment algorithms that provide tools for advanced alignment to pre-existing structures on the sample, as illustrated in Figure 1. Although the algorithm can be used for aligning topside patterns, on their own, they cannot be implemented for backside alignment due to the hidden features. Therefore, in the proposed method, we used the sample corner co-ordinates, which are identifiable from both the top and backside, together with the mapping algorithm of the DLW, to develop this new alignment algorithm of the topside features to the backside co-ordinates.

The known design co-ordinates of features on the samples, such as copper pads, are found by the user and the position is then noted. The layout design file is loaded into the software, and the design co-ordinates are input into the system. Then, the corresponding stage co-ordinates are relocated and input into the system. The software can then calculate how the sample placement differs from the design file by the x- and y-translation (h and k, respectively) and the rotation (θ). The system is then aligned.

By using this alignment software to find h, k, and θ, the design co-ordinates for the corners can also be found by implementing these results into the alignment matrix, as described below. As it is almost impossible to cut the samples to an exact size every time, the alignment matrix is used to correlate the stage and the corresponding design co-ordinates of the corners. This can then be used for any reasonable variation in the size of the sample within the limits of the DLW.

#### 2.1.2. Overview of Steps

The following steps outline the use of the proposed alignment matrix to align the front and backside of the sample with the DLW. The first steps are for using the DLW as normal:

- Prepare the sample with the appropriate alignment marks developed on the topside and load the sample into the DLW stage.
- Load the design file for the pattern of the topside. Note the design co-ordinates of the alignment marks.
- Locate the alignment marks on the sample using the focused camera on the DLW. Make note of these co-ordinates; these are the stage co-ordinates of the alignment marks.
- Locate the corners of the sample; the two opposite corners are best. Note these stage co-ordinates of the selected corners and whether they are top/bottom and left/right.
- Use the Exposure Wizard alignment software (version 4.5.10, Heidelberg Instruments Mikrotechnik GmbH, Heidelberg, Germany) of the DLW. Input the design co-ordinates of the two alignment marks. Find the corresponding alignment marks on the sample, as noted previously. The software will now provide the alignment values of the x- and y-translation (h and k, respectively), and the change in angle, θ.

**Figure 2:**
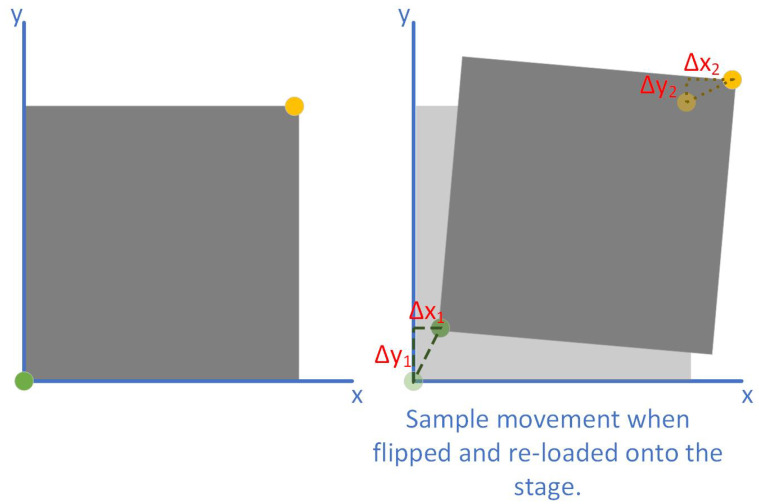
How the co-ordinates of the sample change when it is replaced on the stage. As there is no guarantee of replacing the sample exactly as it was when taking the initial readings, it is expected that there would be a change in the x- and y-directions, as well as a rotation.

Now, the following steps are specifically for the novel method proposed here:

- Input h, k, and θ into the matrix.
- Input the stage co-ordinates of the corners into the matrix. This will provide the design co-ordinates of the corners.
- Remove the sample. Follow the necessary photolithography steps, such as spinning photoresist and any necessary baking. Flip along the y-axis and reload the sample so that the backside is ready for exposure.
- In the alignment software, input the calculated design co-ordinates of the corners. The x co-ordinate will undergo a sign change because of the flip. Figure 2 demonstrates how the reloading effects the stage co-ordinates.
- Locate the corresponding corners using the camera of the DLW and input these into the alignment software. This will give the new alignment data.
- The sample is now aligned, and the exposure can be run. All subsequent photolithography steps can be followed as needed.

### 2.2. Experimental Method

#### 2.2.1. Initial Measurements

The direct laser writer (DLW) used here was the uPG101 from Heidelberg instruments (Heidelberg, Germany), which is a tabletop micro-pattern generator. The alignment accuracy of the system is 200 nm, and the minimum structure size is 0.9 μm, with an address grid down to 40 nm. The laser used is a single-mode diode laser with a wavelength of 375 nm.

Exposure Wizard software is used to control the DLW system, and for running the initial calculations needed for finding the alignment matrix. This allows the user to place a sample onto the stage and focus the camera. There are optical and pneumatic options for this, for the best focus possible.

Before using the DLW, it is important to know the design co-ordinates of the features being used for alignment. These will be compared with their stage co-ordinates to find the design co-ordinates of the corners. Here, alignment marks were used that consisted of four crosses. Only two were needed, and one corner of each cross was used. The marks chosen were opposite (e.g., bottom left and top right) to allow an accurate alignment. These co-ordinates are found from the design file of the top-side pattern on the sample.

Ideally, the sample will have four sharp corners that are easy to locate from both sides of the chip. However, only two, opposite sides are needed, so it is up to the user to decide which corners look the sharpest. One basic way of generating sharper corners when cleaving a silicon sample is to use a diamond scribe along the edge to be cut, but not all the way to the corners. Then, when the sample is cleaved, the corners should have a sharper edge than the scribed section in the middle.

#### 2.2.2. Alignment System

Exposure Wizard software is used to note the stage co-ordinates of at least two corners that are opposite each other, and the corners of at least two of the alignment markers. These can be saved to the ‘position’ list for use later.

As the design co-ordinates of these markers are known, the software can be used to calculate the x- and y-translation (h and k, respectively), as well as the rotation of the design (θ). This is achieved by inputting the design co-ordinates of the alignment marks in the Advanced Alignment option, then locating the matching stage co-ordinates; the software will create the matrix calculations accordingly, providing all three translation and rotation results, which can then be noted.

The matrix calculations can be carried out using this information, following which the sample can be removed and the photolithography steps can start. The photoresist can be spun onto the backside of the sample, and then be replaced onto the stage of the DLW, now with the backside at the top. Here, AZnLOF 2070 (MicroChemicals, Ulm, Germany) was spun onto the sample and baked at 110 °C for 2 min.

The chip can be flipped along the y-axis, and it should be noted that, therefore, when finding the chosen corners, they will change from left to right and vice versa. For example, if the top left and bottom right were chosen and the sample is flipped along the y-axis for backside alignment, the corners will now be the top right and bottom left.

#### 2.2.3. Matrix Calculations

The results from the previous alignment can be added to a matrix that will be used to align the front design to the back.

Reverse engineering means that the x-axis and y-axis shift and the rotation can be calculated to find the difference in the design and stage co-ordinates of the alignment marks. From these alignment results, a matrix can be created to calculate this translation and rotation. An inverse of this matrix can then be applied to the stage co-ordinates of the corners to find the relevant design corner co-ordinates.

When the sample is flipped along the y-axis and placed back on the stage, the x axis will be flipped and so the sign of the x corner co-ordinate also needs to be changed, i.e., the top right corner from the first measurement is now the top left corner.

Now, using both the design and stage co-ordinates, the correct placement for the back pattern can be found by applying this shift to the corners, and the direct laser writer can be aligned and will be ready to expose the photoresist accordingly.

This can be shown in a series of matrix calculations, using translational and rotational shift. A rotation matrix is found with the following form:

(1) $$R=\left[\begin{array}{cc} cos(\theta ) & -sin(\theta )\\ sin(\theta ) & cos(\theta ) \end{array}\right]$$

where θ is the change in the angle.

A translation matrix is found:

(2) $$T=\left[\begin{array}{ccc} 1 & 0 & h\\ 0 & 1 & k\\ 0 & 0 & 1 \end{array}\right]$$

where *h* and *k* are the change in the x- and y-positions, respectively.

When the rotational and translation can be combined into a single matrix and then applied to a set of co-ordinates to determine the new co-ordinates:

(3) $$\left[\begin{array}{c} x^{\prime }\\ y^{\prime }\\ 1 \end{array}\right]=\left[\begin{array}{ccc} cos(\theta ) & -sin(\theta ) & h\\ sin(\theta ) & cos(\theta ) & k\\ 0 & 0 & 1 \end{array}\right]\left[\begin{array}{c} x\\ y\\ 1 \end{array}\right]$$

where *x*’ and *y*’ represent the new x and y co-ordinates, respectively.

Similarly, this function can be reversed to find the original co-ordinates from a new set of position data:

(4) $$\left[\begin{array}{c} x\\ y\\ 1 \end{array}\right]=\left[\begin{array}{ccc} cos(\theta ) & sin(\theta ) & hcos(\theta )-ksin(\theta )\\ -sin(\theta ) & cos(\theta ) & -kcos(\theta )+hsin(\theta )\\ 0 & 0 & 1 \end{array}\right]\left[\begin{array}{c} x^{\prime }\\ y^{\prime }\\ 1 \end{array}\right]$$

Initially, these matrices are found by comparing the design co-ordinates of the alignment features and corners to the stage co-ordinates. This proves the *x*, *y*, and θ offset values.

Once the sample is flipped and re-loaded to the stage, the backside alignment can begin. This uses the newly found design corner co-ordinates as the starting point, and reverse engineers the values of the features that are now hidden. The DLW can then be set to the correct exposure settings, and the final photolithography steps can take place. Here, a post-exposure bake for 1 min at 110 °C took place before the sample was immersed in the AZ 826 MIF developer (MicroChemicals, Ulm, Germany) for 1 min while agitated. Then, the chip was cleaned with DI water and dried with a nitrogen gun before being checked under a microscope to ensure the development was complete. Following successful development, the sample was placed back onto the hotplate at the same temperature for 10 min for a final hardbake.

## 3. Results

To check the reliability of this method, some of the samples, cut from 2 inch silicon wafers, were tested on the topside, to make it easy to check under a normal microscope whether the alignment had worked. Then, the backside alignment was checked, which required an IR camera to image both sides of the sample simultaneously to determine whether the method was successful. The images taken of the topside, which were taken with a microscope, were much clearer and, therefore, easier to analyse.

### 3.1. Topside Alignment

**Figure 3:**
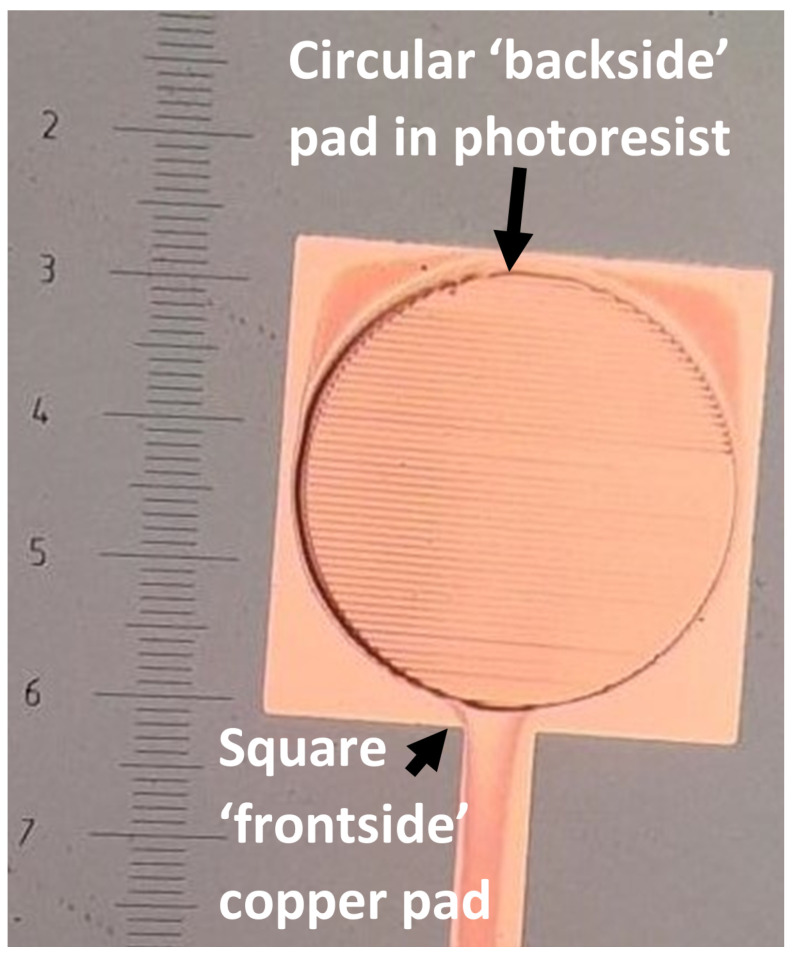
Optical microscope image of the topside sample that was tested using the same design for both the copper liftoff and the photolithography. The square with the line is the copper base, and the circle is the aligned photoresist outline. Ideally, the circle sits centrally within the square. There is very little shift from where the design should be for perfect alignment. A microscope scale is included.

**Figure 4:**
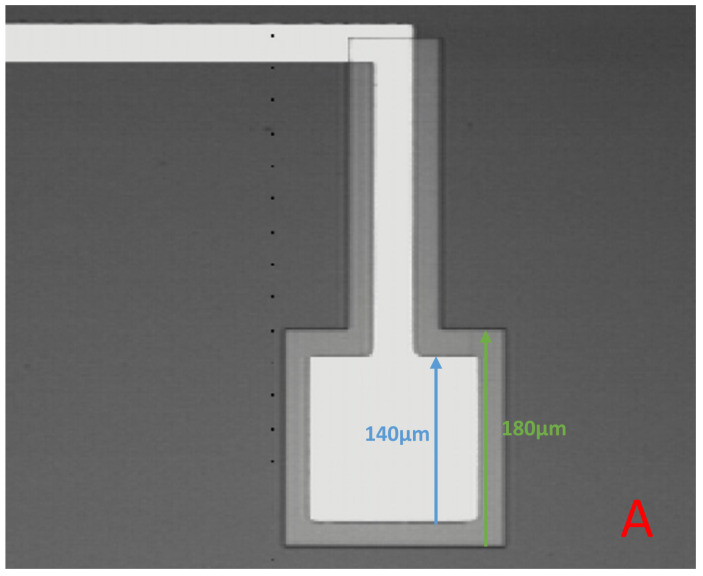

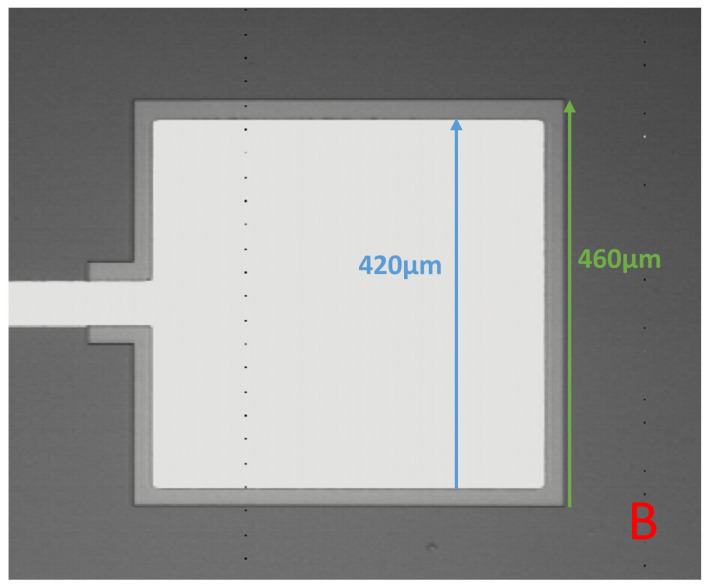
Optical microscope images of the topside sample that was tested using the backside design (flipped along the y-axis to match the topside). The design is clearly well aligned here, providing confidence to proceed with backside samples. In both images, the lightest part shows the topside pattern, with the darker grey area showing the backside pattern. The darkest grey is the silicon background. (**A**) Shows the smallest pad on this design, while (**B**) shows the largest.

Topside samples used the same design for the copper liftoff (to allow a patterned copper layer on the silicon) as for the alignment process. Two designs were tested; the same design already used on the topside (to check the overlap), and the backside design (flipped to match the top now). These can be seen in Figure 3 and Figure 4, respectively. It is clear that this process was successful for both of these designs, so to continue to test the method, a different design was now used, which was identical to the design that would be used for Deep Reactive Ion Etching (DRIE), with the exception that this design was now mirrored horizontally.

#### Analysis of the Topside Alignment

Analysis of the alignment achieved by the topside attempts proved very promising. In Figure 3, it can be seen that the difference in size of the backside pads (on the topside here, seen as a darker, larger pad surrounding the actual topside pad, seen lighter) was 40 μm, expecting a difference of 20 μm in both the x and y directions. An analysis of the topside samples showed a misalignment average of 0.20 ± 0.06 μm and 1.4 ± 0.4 μm in the x- and y-direction, respectively.

### 3.2. Backside Alignment

The accuracy of the technique was checked using an IR camera to image both sides of the chip simultaneously. The backside offered some unexpected added difficulty, in that the corner co-ordinates were slightly more difficult to pinpoint at exactly the same position as the topside, as the width of the sample meant that there may have been a slope from front to back that made it difficult to focus on the same section.

#### 3.2.1. First Iteration: Fully Scribed Samples

**Figure 5:**
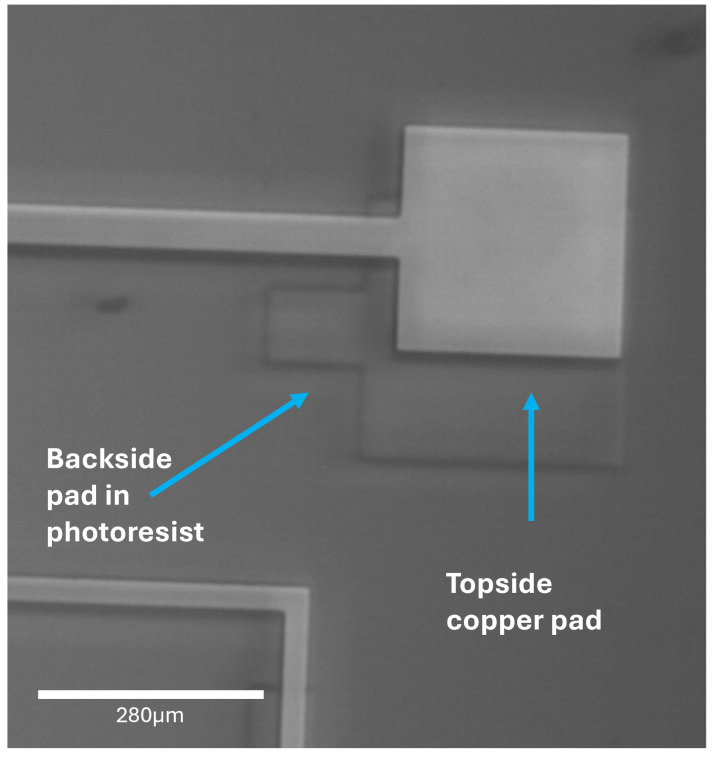
IR camera image of one of the initial tests for the backside alignment. This shows that although the alignment here was not perfect, the topside copper pad did have overlap with the backside photoresist. Further improvements were required for this to be usable.

The samples that were fully scribed showed some initial potential, as seen in Figure 5, with an average x misalignment of 26 ± 11 μm and an average y misalignment of 100 ± 10 μm.

This relates to a percentage misalignment of 17% and 67% for x and y, respectively. The average rotational misalignment was around 0.6 ± 0.2°.

The x misalignment was much lower than the y misalignment throughout. This could be due to the sample being scribed along the y axis, creating rougher edges and, therefore, less clear corners.

#### 3.2.2. Second Iteration: Scribed and Snapped Samples

**Figure 6:**
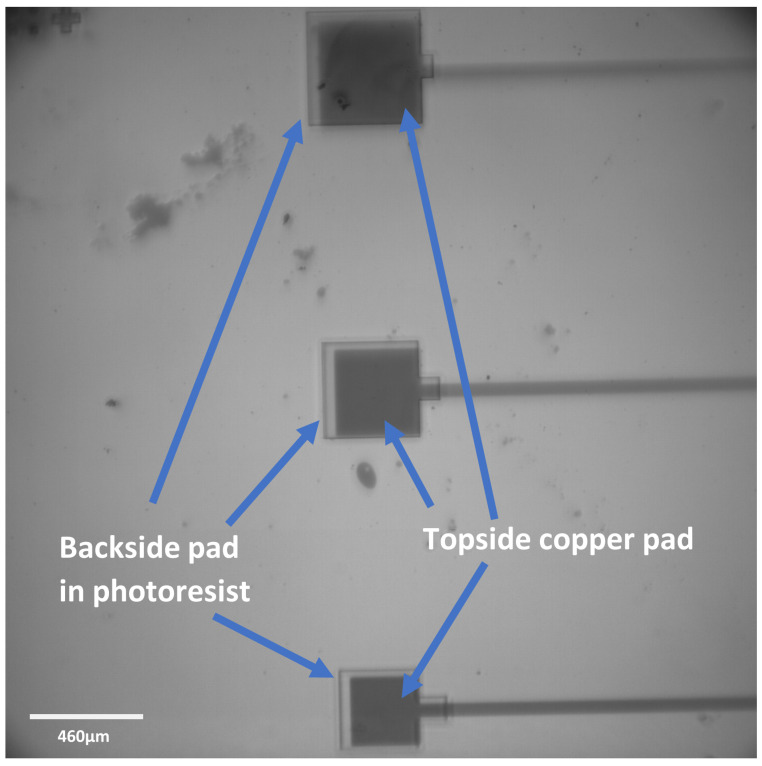
IR camera image of the secondary tests for the backside alignment. This shows that although the alignment here was not perfect, it was good enough for the purpose and the larger backside pad still contained the smaller topside pad.

**Figure 7:**
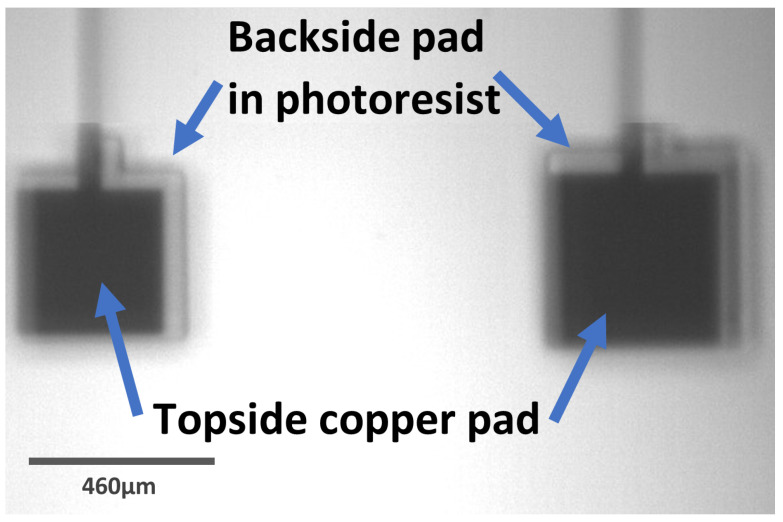
Further IR images of a sample from the second batch that tested backside alignment. The overlay here is not very sharp, making measurement of misalignment difficult to calculate precisely.

Further samples that were created using the novel approach, which involved the cleaving between corners and snapping the sample to keep the corners as sharp as possible, were checked using an IR camera. These images, seen in Figure 6 and Figure 7, were promising, with most being ‘useable’ for many situations, such as for opening heater pads.

These results showed an average misalignment of 23 ± 1 μm in the x-direction, and 8 ± 4 μm in the y-direction. Due to the quality of the imaging, it was difficult to ascertain if any rotational shift occurred.

These results demonstrate an average improvement of up to 3 μm and 92 μm in the x- and y-directions, respectively, as well as an improvement in the associated errors of 10 μm in the x measurements and 6 μm in the y measurements.

The best alignment achieved was within 2 μm of the expected placement.

These almost universal improvements in the alignment demonstrate that with sharper and clearer corners that this method is improved. In particular, the vast reduction in the y misalignment and consistency in these improved values may be attributed to the new snapping method. This method was simple and used equipment found in most microfabrication facilities.

#### 3.2.3. Microheater Samples

**Figure 8:**
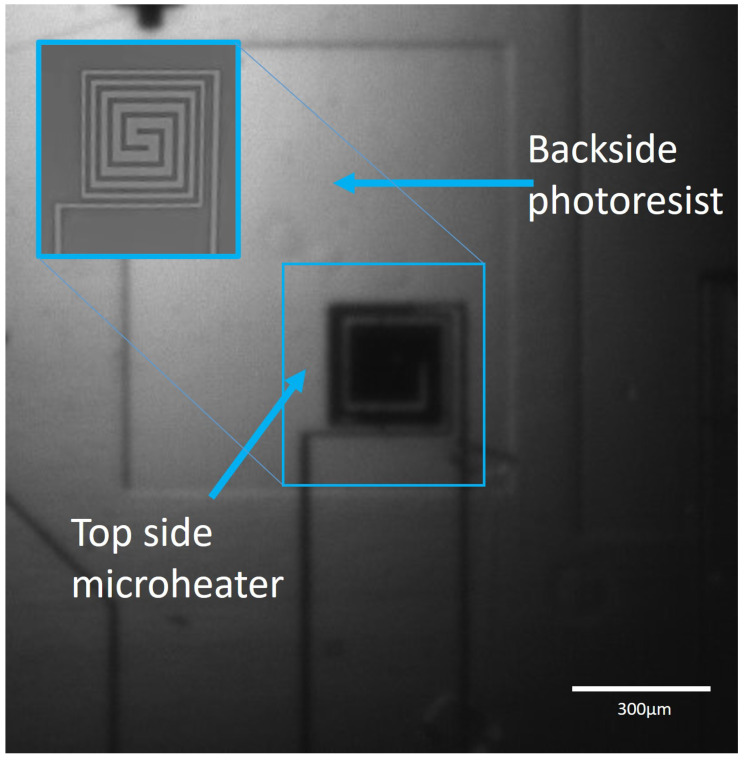
IR image of a microheater sample. The approximate size of the heater is 300 μm × 300 μm. The photoresist on the backside reveals a rectangular area that would be etched to create the membrane, to prevent heat dissipation.

A potential use of this technique is for the back-etching of microheaters on a silicon substrate. To heat an area on silicon without excessive heat dissipation through the substrate requires the backside of the heater to be etched, leaving a thin membrane that the heater is suspended on. This requires an alignment between the front and backside. Here, microheaters of approximately 300 μm × 300 μm were used in varying arrays to test how well the alignment would work with this application. Photoresist was spun onto the backside of these samples, to show where they would be etched for the heaters to sit on the thinner membrane. The results showed that although there was some misalignment, the heater placement was sufficient within the membrane area. These results can be seen in Figure 8.

## 4. Discussion

**Figure 9:**
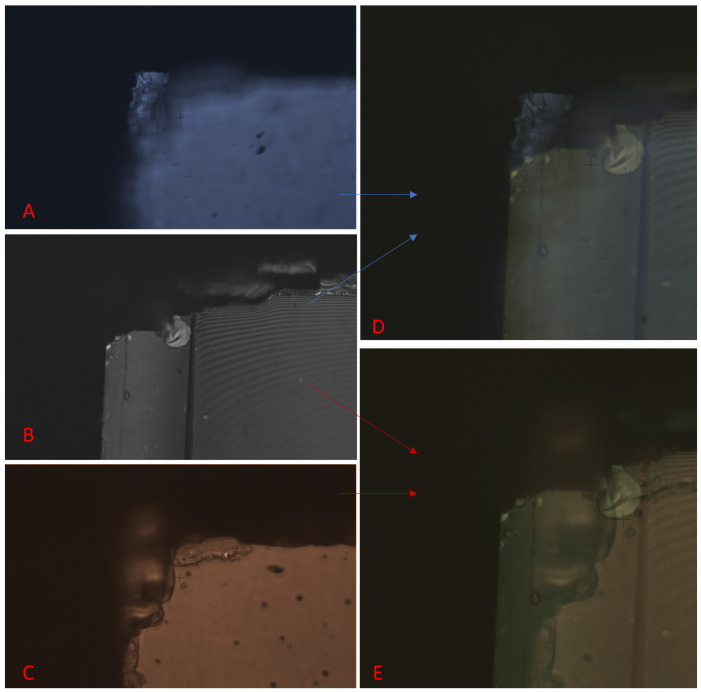
Images from the direct laser writer showing how finding the corner in focus on one side of the sample may be easier than finding that exact same co-ordinate of the corner from the other side. (**A**,**C**) are the topside of the same corner, but with a different focus on the microscope, while (**B**) shows the backside of the same corner. (**D**,**E**) are the result of overlaying (**B**) (backside) with (**A**,**C**) (frontside), respectively.

The difficulties associated with locating the exact corner, and, furthermore, the same location on the front and the back corner, can be seen in Figure 9. What appears to be the clearest and sharpest corner on the topside does not always line up well with where the corner is on the backside. Due to the thickness of the sample and the poor cutting, choosing the exact point to use as the corner co-ordinate can be challenging.

By investigating the front and backside corners of a sample, and the consequent alignment of the features, it is clear that the inaccuracy of the technique and the main source of error are as a result of the difficulty identifying the corners in certain samples. A comparison of the ratio of x:y misalignment between the front and back corner to the ratio of x:y misalignment of the features demonstrates a clear relationship between the two. In some instances, a directly proportional relationship is seen, and in others, the ratio is identical for both the corner selection and the resulting feature misalignment. As it is unknown which optical focus is optimal for any given corner, a clear, straight cut of silicon would produce better results.

### Improvements

The main source of inaccuracy with this method is caused by the difficulties in accurately finding the corners of the sample under the microscope, which is a manual process carried out by the user. As these samples are cleaved manually with a scriber, the lack of visual sharpness in the edges, in particular the corners, can be a source of misalignment, as shown in Figure 9. Out-of-focus edges can become in-focus when the sample is flipped, while the corners that can clearly be seen on the topside would be more difficult to see from the back. This is why good scribing is imperative. In Figure 9, C has a better alignment than A for the backside B, but this would not be clear to the user until the sample has already been flipped. Often, the exact corner co-ordinate is difficult to locate, which can increase misalignment when the chip is then flipped and the corner needs to be located again. Precision cutting methods would help eliminate this issue. The alignment was further improved by extrapolating from the sharper edges to identify the corners. However, this method depended on the chip being loaded onto the stage accurately, as the software provides strictly vertical and horizontal lines for extrapolation.

This was improved upon by only scribing the middle section of the line, leaving the corners uncut, and then snapping the sample to allow the break to follow along the natural grain of the silicon. This showed a definite improvement in the ability to locate the corners accurately and, therefore, resulted in better aligned samples.

This theory of corner inaccuracy is further confirmed by comparing the results found here for backside alignment to the results from the topside alignment; the topside alignment, wherein the same sides of the corners were being used, resulted in significantly better alignment than the same method that was used on two different sides of the same corners.

Further improvements to this may be possible by utilising laser cutting or implementing a dicing saw. This is likely to produce much sharper edges and corners, but is limited not only by access to such machines, but also by the issue of polished samples, which can interfere with the laser used for cutting in some systems. This is, however, still a potential option for single-side polished or unpolished samples.

This method can also be applied to full-size wafers with a flat edge. The points where the flat edge transitions into the curved perimeter can serve as ’corner’ co-ordinates, resulting in a cleaner, sharper edge. This method can then be used for both the full-size wafers and cut samples. It is also possible that this method could be extended to use two orthogonal lines, rather than two corners, which would allow for some flexibility in the sample shape.

This method also has the potential to be used with other substrates, such as FR4, which cannot be used with some traditional double-side mask aligners. As there is no reliance on IR cameras to see both sides simultaneously, this does not limit the material type. Therefore, in an eventuality where backside alignment may be required on PCB, for example when manually routing [15] or in the creation of microfluidic channels [16], this method is superior to using a double-sided mask aligner. It is also scalable to different sample sizes, such as the full-size wafers mentioned previously. The acceptable size range will depend on the individual DLW being used.

Provided the DLW is used for the front and backside photolithography, as is the case here, there should not be any issue with camera or microscope alignment to the stage; however, if this is not the case, then any misalignment between these systems may be amplified. A DLW with a less accurate alignment capability would also be likely to exacerbate any alignment issues already seen here.

## Figures and Tables

**Table 1:** Comparison of the values found in the literature with the novel proposed method, as previously described.

Ref.	Method	Accuracy	Cost	Extra Equipment	Sample Restrictions
[5]	Nikon pre-alignment	500 nm	Very high	Specialist Equipment	None
[6]	Nikon i-line	200 nm	Very high	Specialist Equipment	Silicon only
[7]	EAM	8–11 μm	Low	Small	None
[8]	Mechanical Jig	Theoretical	Middle	Small, made in-house	Size, crystallographic structure
[9]	Jig + Masks	4–5 μm	Middle	Small, made in-house	Size, regular shape
[10]	Projector	1 μm	High	Specialist Equipment	None
[11]	Mask Modification	2–4 μm	Low	Small, made in-house	Flat side of wafer
[12]	Post-Processing Overlay Correction	<1 μm	High	Specialist Equipment	Silicon wafer
[13]	Multiple Exposures	Not listed	Low	None	Silicon wafer
This paper	Our Matrix Method	8–23 μm	Low	None	Sharp corners

## Data Availability

Data curated during this process are available to interested researches. Please contact the corresponding author.

## Abbreviations

The following abbreviations are used in this manuscript:

DLW	Direct Laser Writer
MEMS	Micro-Electro-Mechanical Systems
IR	Infrared
UV	Ultraviolet
EAM	External Alignment Marks
